# A novel exopolysaccharide elicitor from endophytic fungus *Gilmaniella* sp. AL12 on volatile oils accumulation in *Atractylodes lancea*

**DOI:** 10.1038/srep34735

**Published:** 2016-10-05

**Authors:** Fei Chen, Cheng-Gang Ren, Tong Zhou, Yu-Jia Wei, Chuan-Chao Dai

**Affiliations:** 1 Jiangsu Key Laboratory for Microbes and Functional Genomics, Jiangsu Engineering and Technology Research Center for Industrialization of Microbial Resources, College of Life Sciences, Nanjing Normal University, Nanjing 210023, China

## Abstract

Endophytes and plants can establish specific long-term symbiosis through the accumulation of secondary metabolites. Previous studies have shown that the endophytic fungus *Gilmaniella* sp. AL12 can stimulate *Atractylodes lancea* to produce volatile oils. The purpose of this report is to investigate key factors involved in the stimulation of *A. lancea* by AL12 and reveal the mechanism. We identified the active component from AL12 as an extracellular mannan with a polymerization degree of 26–42. Differential membrane proteomics of *A. lancea* was performed by 2D electrophoresis. The results showed that there were significant differences in the expression of 83 proteins. Based on these results, we conclude that AL12 secreted mannan contributes to the antagonistic balance seen in interactions between AL12 and *A. lancea*. One portion of the mannan was degraded to mannose for hexokinase activation, promoting photosynthesis and energy metabolism, with a potential metabolic fluxes flowing towards terpenoid biosynthesis. The other portion of the mannan directly enhanced autoimmunity of *A. lancea* through G protein-mediated signal transduction and the mannan-binding lectin pathway. Volatile oil accumulation was ultimately promoted in subsequent defense reactions. This study provides a new perspective on the regulation of secondary metabolites by endophytic fungal elicitors in medicinal plants.

In nature, plants maintain a variety of relationships with different microorganisms, including mutualism with endophytes or antagonism with pathogens. Endophytes are usually characterized by the fact that they do not cause harm to the host^1^. Indeed, some endophytes, such as mycorrhizal fungi or other growth promoting endophytes can be beneficial to plants^2^. Unlike pathogens, endophytes do not cause strong hypersensitivity reactions in the host. Typically, rhizobia can modulate and optimize the host legume’s susceptibility to infection^3^. But beyond that, long-term colonization can also induce the accumulation of various secondary metabolites in hosts^4^. Schulz *et al*. suggested that the relationship between endophyte and host plant can be explained by the balance antagonism hypothesis^5^. The secondary metabolites produced by the host plant are likely to be the key substances responsible for the maintenance of the antagonistic balance between endophyte and host plant. This is mainly reflected in two aspects: On the one hand, endophyte overgrowth can be restricted to a certain extent by the synthesis of plant secondary metabolites, suppressing the invasion of endophytes in the host; On the other hand, the synthesis of these secondary metabolites also can weaken the host’s own defense system. Although the mutualistic symbioses between endophytes and host plants can be explained by the antagonistic balance hypothesis, there is still a lack of direct evidence about how secondary metabolites are used to maintain the delicate balance between endophytes and host plant, and especially the specific mechanisms in which these biological molecules are involved.

Recently the effects of endophytes on the accumulation of active ingredients by medicinal plants have received increasing attention^4^. *Atractylodes lancea*, also known as CangZhu in China, belongs to the *Asteraceae* family of plants. Its rhizome, commonly called Rhizoma Atractylodis is used in China as an important crude drug against rheumatic diseases, digestive disorders, night blindness and influenza. In other Asian countries, such as Korea and Japan, pharmacopoeias have also recorded this herb as part of traditional diuretic and gastric prescriptions^6^. Recently, a study has shown that *A. lancea* extracts have various pharmacological activities including lipase inhibition and antiobesity effects^7^, anticancer, anti-hypertensive, anti-platelet, anti-ulcer, anti-inflammatory, antimicrobial, and antipyretic activities, as well as activity on the nervous and gastrointestinal systems^8^. One of the main efficacious components of *A. lancea*, β-eudesmol, can be used as a potential molecule for therapy in mast cell-mediated inflammatory diseases^9^. In recent years, many researchers have used increasingly sophisticated techniques to study *A. lancea* and have found the main medicinal active ingredients of *A. lancea* were volatile oils, whose principal components include sesquiterpenes and polyacetylene secondary metabolites, such as β-caryophyllene, zingiberene, β-sesquiphellandrene, caryophyllene oxide, hinesol, β-eudesmol, atractylone, and atractylodin^10^.

However, the natural reserves of *A. lancea* have sharply decreased due to a variety of exploitative practices. Currently, tissue culture derived plantlets of *A. lancea* are available for large-scale cultivation. However, a key question is how to maintain or improve herb quality when the plants are cultivated away from their native environment. In addition to a number of inorganic environmental factors that can be controlled under tissue culture conditions, endophytes are considered to be important biological factors that can simulate part of the original habitat of host plants. Our research group is committed to solving this problem through the use of endophyte-based approaches. We have suggested that endophytes can promote the accumulation of volatile oils and improve their quality. We have succeeded in obtaining several endophytes that can establish a good relationship with *A. lancea*. We also found that these endophytes and their elicitors can effectively stimulate volatile oil accumulation. In particular, a systematic investigation was performed to investigate the effects of an endophytic fungus *Gilmaniella* sp. AL12 and its crude elicitor on the defense and metabolic responses of *A. lancea*. It was shown that both the fungus and elicitor led to an enhancement of volatile oil accumulation in *A. lancea*^11^,^12^. However, it is still unclear how the accumulation of secondary metabolites is influenced during host-endophyte interactions. Also, the specific active component of the endophytic fungus that enables the interaction and the molecular mechanism behind these interactions are still unclear.

Plant endophyte elicitors are signaling molecules derived from a fungus or bacterium. These elicitors can not only induce plant disease resistance, but also promote the plant to produce various secondary metabolites, such as terpenoids, alkaloids, saponins, phenols and flavonoids^3^. Plant endophyte elicitors are mainly cellular surface structures or secretions, including polysaccharides, polypeptides, glycoproteins and unsaturated fatty acids^13^. Polysaccharides, as a type of elicitor, are mainly cell wall components that are often derived from digestion of the endophyte cell wall by plant hydrolases. Their structures are mainly composed of heteropolysaccharides^14^. However, the complex interactions between endophytes and host plants sometimes rely on certain extracellular polysaccharides secreted by endophytes^15^. In the interaction of plants and endophytes, it is still unclear whether endophyte elicitors (extracellular polysaccharides) are specific or common for the host plant.

The purpose of this study was to identify the active structural material of the endophytic fungus *Gilmaniella* sp. AL12, and then use this as a basis to further identify host plant membrane proteins which respond to this material. Here, we have identified and isolated the elicitor. Differential membrane proteome analysis of *A. lancea* after treatment with the *Gilmaniella* sp. AL12 elicitor was performed by 2D electrophoresis. This study will provide a basis for the use of endophytic fungal elicitors to promote the accumulation of active ingredients in medicinal plants.

## Results

### The effect on volatile oil biosynthesis by different elicitor components of AL12

Our previous studies showed that the endophytic fungus *Gilmaniella* sp. AL12 and its sterilized crude elicitors could significantly stimulate volatile oil accumulation in *A. lancea* plantlets^12^. To further determine the type and physiological sources of AL12 elicitors that facilitate volatile oil accumulation in host plants, the various components were classified into different groups. Sterilized crude AL12 mycelium (Component A), chitinase and β-1,3-glucanase hydrolyzed cell wall products of AL12 (Component B), and crude extracellular polysaccharides from an AL12 fermentation broth (Component C) were obtained. Changes in the concentration of volatile oils and eight main components (β-caryophyllene, zingiberene, β-sesquiphellandrene, caryophyllene oxide, hinesol, β-eudesmol, atractylone, and atractylodin) were observed in *A. lancea* plantlets after injection with various AL12 components (Table 1).

The results showed that 20 days after treatment with the different components, the crude extracellular polysaccharides (Component C) stimulated the production of volatile oils to near the level seen when crude elicitors (Component A) were used, and the total production of volatile oils was increased significantly (1.69-fold higher than the control). The changes in the amounts of oxygenated volatile oils, for example caryophyllene oxide, hinesol, β-eudesmol, atractylone and atractylodin, were particularly remarkable. The latter four types of secondary metabolites are commonly believed to be important genuine ingredients in *A. lancea*. However, the degraded mycelium products (Component B) did not appear to increase volatile oil accumulation. These results do not match with the results of other reports that suggest that the cell wall degradation products of endophytic fungi were suitable elicitors^14^. Most compounds in Component A were intracellular, and these complex materials are unlikely to be released from the cells during an actual symbiosis due to the natural barrier provided by the cell wall. We speculated that certain extracellular polysaccharides in Component C were the most likely active ingredients for volatile oil accumulation in *A. lancea*.

### Purification and identification of the AL12 exopolysaccharide elicitor

To determine which specific compound from the AL12 extracellular polysaccharides was responsible for stimulating the accumulation of volatile oils in *A. lancea*, it was necessary to fractionate the crude polysaccharide mixture. The AL12 crude polysaccharide (Component C) was dissolved in water, after degreasing, the Sevag method and molecular sieve gel chromatography were used to remove proteins and other impurities. The polysaccharide fraction was collected (Fig. S1) and used for ultrafiltration, after which the retentate was further purified by ion-exchange chromatography. The polysaccharide solution was then further purified by active carbon filtration and dialysis. Exopolysaccharide purity was evaluated by gel permeation chromatography (GPC) (Fig. S2). The purified polysaccharide was finally obtained as a white powder after freeze-drying for storage and further use.

The hydrolyzed monosaccharide derived from the purified extracellular polysaccharide was compared against analytical monosaccharide standards by thin layer chromatography (TLC). The same Rf value was seen with the hydrolyzed monosaccharide (0.29) and mannose (0.29), and their colors were also similar (dark blue) (Fig. S3). Because both the Rf values and colors of the glucose and mannose monosaccharides were close, it was necessary to further verify the identity of the monomer. The hydrolyzed monosaccharide derivative was shown to be a single mannose by GC-MS analysis (Fig. S4). Therefore, the AL12 extracellular polysaccharide was a mannan as identified by TLC and GC-MS analyses.

**Figure 1:**
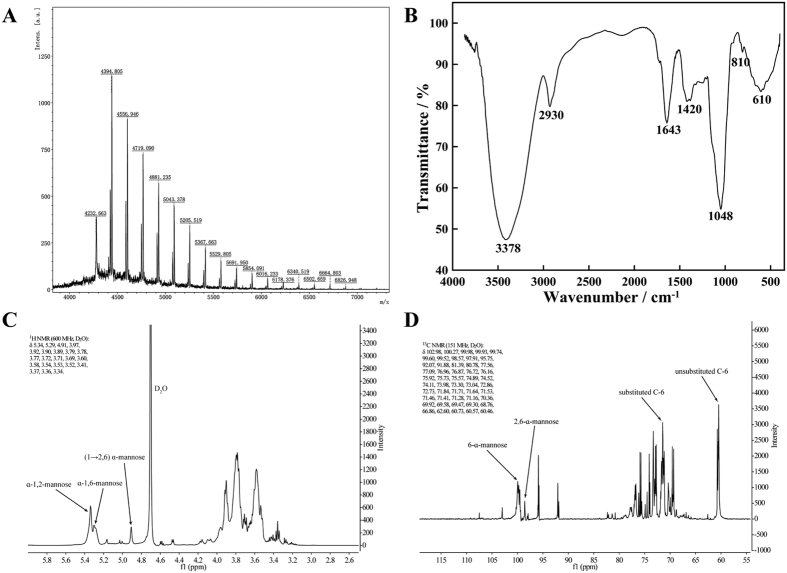
Figure 1 The identification of *Gilmaniella* sp. AL12 exopolysaccharide elicitor. (**A**) Degree of polymerization as determined by MALDI-TOF MS; (**B**) FT-IR spectrum; (**C**) ^1^H NMR spectrum; (**D**) ^13^C NMR spectrum.

The MALDI-TOF MS spectrum of the AL12 extracellular polysaccharide confirmed the aforementioned analyses (Fig. 1A). The repeating peaks at equal intervals of 162.14 Da suggested a molecule composed of structural units with a molecular mass of 162.14 Da, suggesting that the extracellular polysaccharide was a homopolymer. We assumed that the polysaccharide was a linear molecule produced by the dehydration of water, and the molecular weight of the monomer would then be 180.16 Da (162.14 + 18.02), meaning that the molecular weight of the polysaccharide could be calculated from the formula 180.16 × n − 18.02 × (n − 1), where n is the number of residues. The polysaccharide had a series of (M + H)^+^ values that fit the formula. For example, the signal at 4232.663 was consistent with the value obtained by substituting 26 for n in the above formula. The molecular mass of mannose is 180.16 Da. We speculated that the polysaccharide was mannan composed of between 26 to 42 mannose monomers.

The purified mannan was characterized by infrared spectroscopy (FT-IR) and nuclear magnetic resonance spectrometry (NMR) to determine the primary structure. The absorption bands shown in Fig. 1B were typical for the FT-IR spectrum of a polysaccharide. The strong absorption peak at 3378 cm^−1^ represents O-H stretching vibration of the sugar-ring −OH group, the peak at 2930 cm^−1^ results from stretching vibration of C-H, the peak at 1643 cm^−1^ is a flexural vibration peak of O-H, the peak at 1420 cm^−1^ is angular vibration of C-H, the strong absorption peak at 1048 cm^−1^ represents C-O stretching vibration of sugar ether linkage (C-O-C), the peak at 810 cm^−1^ is characteristic for the pyran configuration of α-mannose, while the peak at 610 cm^−1^ comes from stretching vibration of pyranose skeleton symmetry. Thus, the AL12 extracellular polysaccharide could be considered an α-pyran mannan.

The aforementioned putative mannan structure was further verified by NMR. ^1^H NMR spectrum (Fig. 1C) of the polysaccharide contained three signals at 4.91, 5.29 and 5.34 ppm in the anomeric region. The anomeric proton signals at 4.9–5.5 ppm were assigned to the α-configuration of the pyranose units. The analysis was consistent with the FT-IR results. The H-1 signal at δ 5.34 ppm had a close proximity to δ 5.3 ppm for α-1,2-mannose side chain residue of mannan; the δ 5.29 ppm was a characteristic peak of α-1,6-mannose main chain residue of mannan; and the signal at δ 4.91 ppm was the (1→2,6)-linked α-mannopyranose residue. Other proton signals were located in the region of 4.0–3.3 ppm, which were assigned to H2–H6 of the sugar residues.

In the ^13^C NMR spectrum (Fig. 1D), a chemical shift occurred at δ 98–103 ppm, meaning that the C-1 bond was replaced and all of the sugar residues have an α-mannoside bond. The anomeric carbon signals at δ 99.52 and 100.27 ppm were characteristic peaks for 6-α and α-2,6-mannose residues of mannan. There was no resonance signal at δ 82–88 ppm, meaning that all the sugar residues were pyranoid glycosides. As there were some resonance signals at δ 76–80 ppm, the molecule contains many substitution linkages on C-2, C-3 and C-4. There were several resonance signals at δ 70–76 ppm, indicting free bonds at C-2, C-3 and C-4. There were several chemical shifts near 75–78 ppm that showed that the C-5 bond was unsubstituted. There was an obvious chemical shift close to δ 70 ppm, indicating that C-6 substitution had occurred. However, the chemical shifts near 60 ppm showed that a few C-6 bonds were not yet unsubstituted. According to the ratio of peak height, the following conclusions can be drawn about the specific structure of the mannan: backbone substitution occurs between the C-1 and C-6 bond, which is α (1→6), and the branched glycosidic bonds are α (1→2), α (1→3) and α (1→4).

Bases on the above comprehensive analysis by TLC, GC-MS, MALDI-TOF MS, FT-IR and NMR, the structure of AL12 extracellular polysaccharide is a novel α-1,6-pyran mannan homopolysaccharide (the branched glycosidic bonds including α (1→2), α (1→3) and α (1→4).) with a polymerization degree of 26–42. To the best of our knowledge, the structure of secreted mannan reported in this paper is completely different from the common fungal cell wall mannan.

### Experimental evidence for extracellular mannan as an elicitor

In order to verify that mannan is truly an effective stimulator of volatile oil accumulation in *A. lancea*, purified mannan was applied to *A. lancea* plantlets. The results showed that application of the purified mannan increased volatile oil production, and the total volatile oil amount was also increased compared to when the crude polysaccharide was applied to the plantlets (Table 1). This could mean that other components of the crude polysaccharide mixture are not able to serve as elicitors, so the induction effect of mannan was fully exploited after their removal. This experiment confirmed that the elicitor is mannan, as identified from Component C. Microscopic observation^15^ also showed that the size of oil cavities for volatile oil accumulation became significantly larger after treatment with purified mannan, compared with the control (Fig. S5).

**Figure 2:**
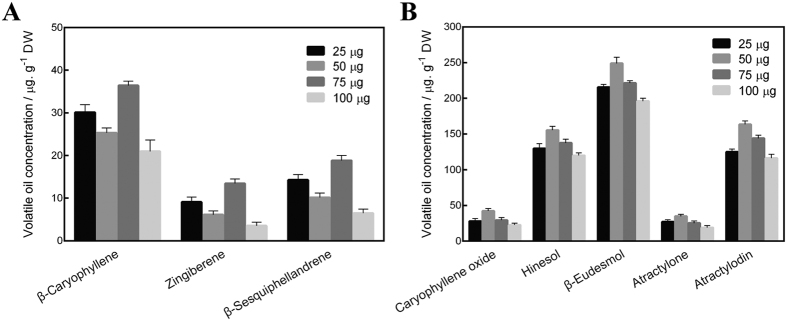
Variation in the levels of eight volatile oil components after treatment with different concentrations of mannan (wither 25, 50, 75 or 100 μg per plantlet). (**A**) Three types of oxygen-free volatile oils; (**B**) Five types of oxygenated volatile oils.

We also further studied changes in the amounts of eight volatile oils after spraying four different amounts (25, 50, 75 and 100 μg) of purified mannan (Fig. 2). 50 μg was used to simulate the *in vivo* secretion of mannan when a symbiotic relationship was established between AL12 and *A. lancea*. As shown in Fig. 2A, compared with the 50 μg mannan treatment, the oxygen-free sesquiterpenoid content increased significantly after treatment with either 25 μg or 75 μg mannan. However, the sesquiterpenoids content was lowest after 100 μg mannan treatment. Interestingly, the content of oxygenated sesquiterpenoids and polyacetylene compounds differed greatly from the above changes, with the oxygenated volatile oil content highest after treatment with 50 μg mannan (Fig. 2B). These results show that the main role of mannan is to promote the accumulation of oxygenated volatile oils. There is likely to be an optimal concentration of the mannan for this promoting effect, therefore, we speculate that a dose of 50 μg mannan produced by AL12 was the optimal effectiveness in *A. lancea*. From the above experiment, other results can be inferred: Three types of oxygen-free sesquiterpenoids are likely to be the precursors of oxygenated volatile oils, as oxygen-free sesquiterpenoids increased with increasing mannan dose, and these were eventually consumed and converted into oxygenated volatile oils at the optimal 50 μg mannan. When the quantity of applied mannan was more than 50 μg, the conversion rate of oxygen-free sesquiterpenoids decreased and they accumulated (75 μg mannan treatment), eventually preventing the biosynthesis of all volatile oils due to overreaction caused by too much applied mannan (100 μg mannan treatment).

**Figure 3:**
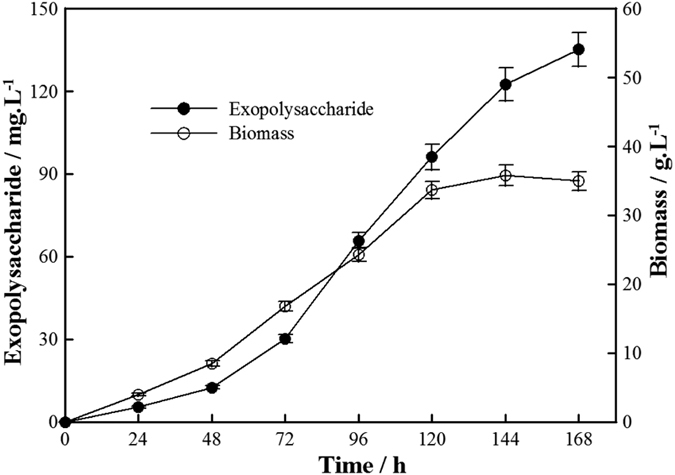
Biosynthesis curve of exopolysaccharides produced by *Gilmaniella* sp. AL12 in 0.5 L shake flask.

In addition, we performed a fermentation experiment of the fungal AL12 strain *in vitro*. The results showed that mannan was secreted into the fermentation broth as the cells grew (Fig. 3). These results suggest that the polysaccharide is not a structural component released from fungi cell wall degradation, but is a specifically synthesized metabolite. We hypothesize that the long-term symbiosis between AL12 and *A. lancea* has allowed these organisms to establish an interacting relationship through the secretion unique extracellular mannan.

### Differential membrane proteomics of *A. lancea* in response to mannan elicitor

In order to identify potential changes in protein abundance levels in response to the exopolysaccharide elicitor, differential membrane proteomic profiles of *A. lancea* plantlets treated with or without the purified extracellular mannan were compared by two-dimensional gel electrophoresis (2-DE). In this study, we investigated the membrane proteome of *A. lancea* as membrane proteins have been previously shown to be significantly affected by external stimuli in plants^16^. Beyond this, we also speculated that proteins would be most likely to interact with mannan would be involved in signal transduction pathways, and these signal transduction proteins are primarily located in cell membrane.

**Figure 4:**
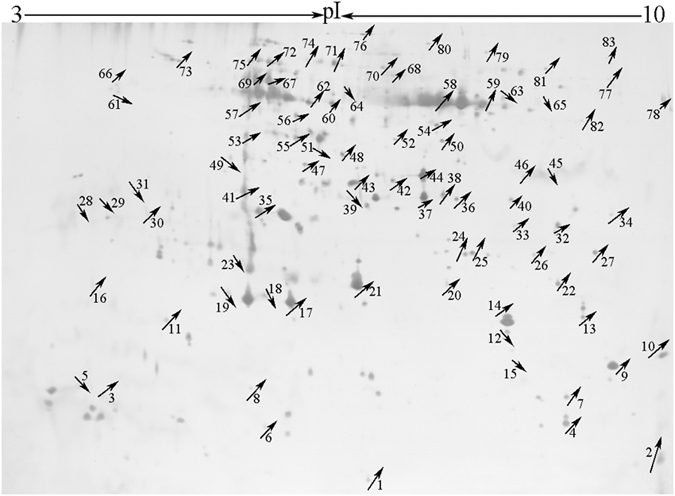
Comparative proteome map of *A. lancea* after exposure to *Gilmaniella* sp. AL12 exopolysaccharides (up arrow, up-regulated proteins; down arrow, down-regulated proteins).

After optimization of the 2-DE gels, approximately 248 proteins were resolved between pH 3 and 10, of which 83 proteins were identified that differed by more than 2-fold (Fig. 4). These proteins (66 up-regulated spots and 17 down-regulated spots) were all selected for identification by MALDI-TOF MS/MS. The selection of these proteins was based on abundance levels (fold change >2) and the position of the spots. Only those spots that could be excised without the risk of cross contamination were selected for identification. The identities of the selected proteins obtained by MS are shown in Table 2. When the theoretical and observed Mr/pI values of the identified proteins were compared, a discrepancy between the theoretical and observed Mr/pI values was observed. This discrepancy occurs frequently in proteome research and appears to be mainly caused by post-translational modifications of proteins, such as proteolytic processing, glycosylation, and phosphorylation^17^. However, it was also possible that the protein spots detected were merely protein fragments.

**Figure 5:**
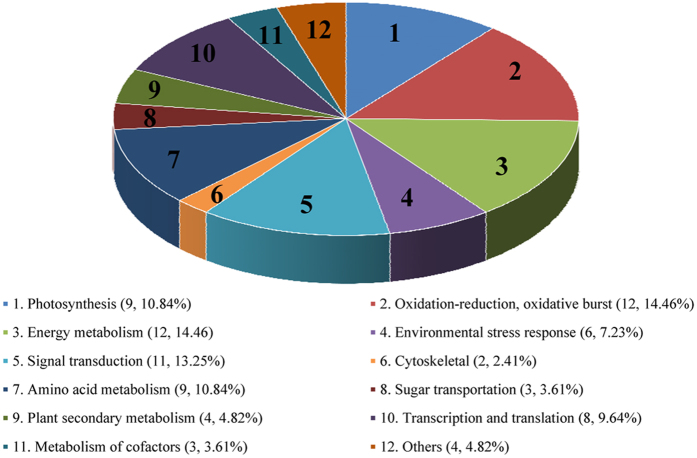
Functional classification of the 83 identified proteins. The proteins were assigned to 12 functional categories (no multiple assignments) according to ExPASy and Gene Ontology database information.

As shown in Table 2, the expression levels of 83 proteins were significantly different between the mannan elicitor treated group and the control plantlets. The majority of the identified proteins were membrane proteins, indicating that the strategy we adopted for membrane protein extraction and 2-DE was reliable. The differentially expressed proteins were classified into twelve functional categories, such as: photosynthesis; oxidation-reduction, oxidative burst; energy metabolism; environmental stress response; signal transduction; cytoskeletal; amino acid metabolism; sugar transportation; plant secondary metabolism; transcription and translation; metabolism of cofactors; and others (Fig. 5).

The expression of photosynthesis-related proteins including chlorophyll a/b binding protein and ribulose bisphosphate carboxylase were all up-regulated. This is consistent with our previously reported results, where the colonization of *A. lancea* by the endophytic fungus AL12 improved the rate of photosynthesis and increased the content of chlorophyll and soluble sugars. Several plant secondary metabolism-related proteins were found to be up-regulated, such as oxysterol-binding protein, cytochrome P450, and epoxide hydrolase, which is consistent with the physiological phenomenon of polysaccharide elicitors significantly improving the yield of volatile oils in *A. lancea*. The enhancement of photosynthesis led to a direct up-regulation of all energy synthesis-related proteins (ATP synthesis enzymes). This enhancement of energy metabolism activity would increase the amount of ATP, which combined with the enhancement of redox activity (redox enzymes depend on NADH/NAD), would promote growth and secondary metabolic activity of the host plant. The enhancement of redox activity is necessary for the biosynthesis of oxygenated sesquiterpenes.

The increased activity of the redox and oxidative burst pathways (e.g. monooxygenase enzymes) can induce the production of the signal molecule H_2_O_2_. Our previous studies have shown that AL12 elicitors can promote the accumulation of volatile oils through H_2_O_2_-mediated signal transduction in *A. lancea*^2^. Together with the current round of experiments where we saw the up-regulation of defense response-related proteins, we can conclude that the secretion of mannan by the endophytic fungus AL12 initiated a series of defense responses in *A. lancea*. In order to avoid damaging itself by these defense responses, *A. lancea* would synthesize large amounts of oxygenated sesquiterpenes, which form a major component of the volatile oil produced by the plant. This is similar to in nature to the effect of abiotic stress, such as the link between the wounding response and plant secondary metabolite production^18^. However, a more important question is how mannan produces this effect in *A. lancea*, or how do these elicitors act in the signal transduction pathway? Fortunately, we have identified a number of signaling components closely related to mannan or mannose, such as G-protein signaling pathway proteins, hexokinase and mannan-binding lectin.

### Verification of protein expression by qRT-PCR

**Figure 6:**
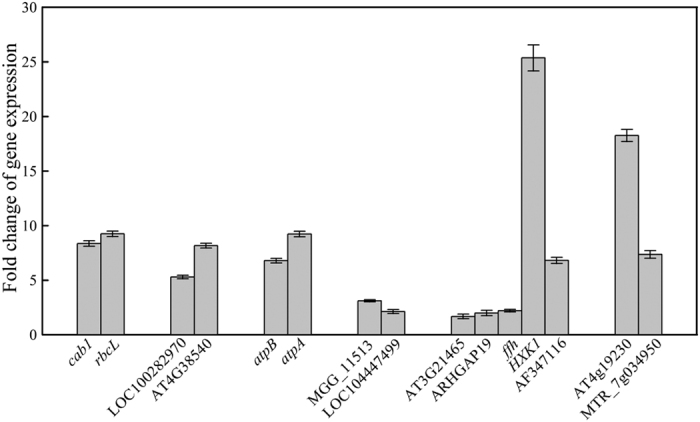
Changes in gene expression levels of fifteen proteins in *A. lancea* after treatment with *Gilmaniella* sp. AL12 exopolysaccharides. The key genes of related pathways: (1) Photosynthesis pathway: *cab1*, *rbcL*; (2) Oxidation-reduction and oxidative burst pathways: LOC100282970, AT4G38540; (3) Energy metabolism: *atpA*, *atpB*; (4) Stress response: MGG_11513, LOC104447499; (5) Signal transduction pathway: AT3G21465, ARHGAP19, *ffh*, *HXK1*, AF347116; (6) Terpenoid metabolism: AT4g19230, MTR_7g034950. The values are from three independent experiments.

In order to confirm the reliability of the proteomic results, the gene expression levels of fifteen proteins with the greatest differences between untreated and treated groups were verified by qRT-PCR (Fig. 6). Chlorophyll a/b binding protein (*cab1*) and ribulose-1,5-bisphosphate carboxylase/oxygenase (*rbcL*) are key photosynthetic enzymes and are crucial for plant growth. NADP-dependent oxidoreductase (LOC100282970) and monooxygenase (AT4G38540) are key proteins of oxidation-reduction and oxidative burst pathways. ATP synthase beta subunit (*atpB*) and ATP synthase CF1 alpha subunit (*atpA*) mediate energy balance. Heat shock protein SSB1 (MGG_11513) and putative disease resistance protein RGA1 (LOC104447499) are closely related to the stress response. Adenylyl cyclase (AT3G21465), rho GTPase-activating protein 19 isoform X5 (ARHGAP19), GTP binding signal recognition particle protein (*ffh*), hexokinase (*HXK1*), and mannan-binding lectin (AF347116) are proteins that are involved in signal transduction and are likely to participate in mannan-mediated stimulation of volatile oil biosynthesis. The cytochrome P450 (AT4g19230) and epoxide hydrolase (MTR_7g034950) are important proteins related to plant terpenoid metabolism^19^. These proteins most likely promote volatile oil accumulation by modulating *A. lancea* metabolism. As shown in Fig. 6, the expression levels of all 15 genes were significantly increased in the exopolysaccharide treated plantlets, consistent with the proteomic results.

## Discussion

In this paper, a novel extracellular mannan was identified from a plant endophyte that could significantly stimulate secondary metabolite production in the host. The present study showed that plant endophyte elicitors can be derived from live endophytic fungi, extracts of fungal suspensions or mycelia, cell wall degrading ingredients, and the soluble components from mycelia high temperature treatment or partial acid hydrolysis. The majority of elicitors are polysaccharides, glycoproteins, proteins (or polypeptides), and unsaturated fatty acids^20^,^21^. However, as a large number of polysaccharide elicitors have been identified, these are the best studied. Polysaccharides are mainly used as a structural component of the cell wall and are usually released when the fungal cell wall has been degraded. Polysaccharides also act as signaling molecules and play a role in plant defense response of the host, and the process of growth and development^14^. For pathogens, the production of polysaccharide elicitors generally causes an allergic reaction in the host plant and alters the metabolic pathways of plant cells, leading to the synthesis of secondary metabolites, which can then act on the pathogens to prevent further invasion. For endophytes, polysaccharide elicitors would act as “antagonism balancers” between the host plant and the endophyte, promoting a relationship of coexistence. Kim *et al*. reported and identified a cell wall carbohydrate isolated from *Leptosphaeria maculans* that contributes to the defense response of the host, *Brassica napus*^22^. Furthermore, hydrolytic cell wall polysaccharides from the endophytic fungus *Fusarium oxysporum* Dzf17 can stimulate the accumulation of the secondary metabolite diosgenin in the host *Dioscorea zingiberensis*, and the maximum yield post-induction was three times that of the control^23^.

As polysaccharides are a structural component of the cell wall, universal representatives of this class of elicitors include dextran, chitosan, chitin and their derivatives. The plant *Glycine max* L. exploits a specific β-dextran, a 1,6-β-linked and 1,3-β-branched heptaglucoside, present in cell walls of the oomycetal pathogen *Phytophthora sojae*, as a signal compound that elicits the onset of defense reactions^24^. Chitosan is a broad-spectrum elicitor that can act on many plants^25^. Currently, a number of receptor proteins in close combination with acetyl chitosan were found in the plasma membrane of some plants including wheat, barley, and carrots^26^. Chitin has been extensively studied as a model elicitor in many plants^27^. Most polysaccharide elicitors found in the cell wall of fungi do not seem to have particular specificity. Poly- or oligo- saccharides of the cell wall are the most well studied signaling molecules in elicitor-based signal transduction. Many elicitors, such as chitin, xyloglucans, chitosan, β-glucan and oligogalacturonide, exhibit elicitor activity across different plant species and induce plant defense reactions and secondary metabolite accumulation, suggesting that different plants possess common receptors to sense these signals.

There is little information regarding the role of extracellular polysaccharides in interactions between endogenous microbes and host plants. These extracellular polysaccharides are also structurally weaker than those present in the cell wall^28^. We propose that the difference between extracellular polysaccharides and cell wall polysaccharides is that the role of former is specific for mediated host-endophyte interactions, and this specificity imposes certain requirements on the polysaccharide structure itself. There are many examples of mannan-based elicitors in previous studies^29^,^30^,^31^,^32^,^33^, their structures mainly be divided into two categories: The first is a mannan oligosaccharide usually derived from the hydrolysis of locust bean gum in *Ceratonia siliqua*, which has a degree of polymerization of 5–8^30^,^31^. The second structure is an N-containing mannan from the yeast cell wall, which belongs to the class of glycopeptides^29^,^32^,^33^. However their structures are different to the mannan elicitor reported in this paper. In the previous text stated that the unique mannan secreted by endophytic fungus AL12 is the main stimulator of volatile oil accumulation in *A. lancea*. In consequence, we can conclude that unique structure of mannan elicitor is critical influencing factor for host plant.

We extracted membrane proteins from *A. lancea* and identified 83 differentially expressed protein spots by 2-DE, which were assigned to 12 functional categories. This is the first time that membrane proteomics has been applied to a medicinal plant. As has already been mentioned, in terms of biochemical pathways, the mannan elicitor promoted photosynthesis, redox active response activities, and accelerated energy metabolism, forming a larger proton electrochemical gradient, which will help more of the elicitor enter the cell, promoting a positive feedback loop^30^. In addition, the mannan elicitor can stimulate oxidative burst, promoting large-scale synthesis of volatile oils by H_2_O_2_-mediated pathway, which is in agreement with previously reported experimental results^2^. A similar effect is seen by stimulation of oxidative burst in *Linum austriacum* by a heteropolysaccharide mannan from yeast, which leads to lignan accumulation^32^. However, this process is different from the inhibition of reactive oxygen burst by a small molecule mannan (the degree of polymerization of the yeast heteropolysaccharide is 5–8)^29^. The main components of *A. lancea* volatile oil that were induced by the mannan elicitor in this study were mostly oxygenous sesquiterpenes. To the best of our knowledge, the oxidative burst can promote accumulation of oxygenous sesquiterpenes. Our group has reported that the synthesis of oxygenous terpenoids in plants is not always the result of an enzymatic reaction, but may also be derived from stress responses to external reactive oxygen species^34^. We conclude that endophytic fungus AL12 plays a very important role in the synthesis and diversity of oxygenous sesquiterpenes in its host *A. lancea* through the production of mannan.

Although several studies have emphasized that mannans function as elicitors primarily by activating defense responses of the host plant, there are no direct reports that have investigated the specific pathways through which they exert their effects. According to the central dogma of environment information: environmental stimulation *in vitro* leads to regulation of gene expression and physiological and biochemical reactions, which change the phenotype of an organism. Based on this dogma, we can speculate that the mannan elicitor may play a role as both an *in vitro* stimulating factor of *A. lancea*, and as a sugar signaling molecule. It has been reported that sugars have a role as primary messenger-like hormones in plants^35^,^36^,^37^. Studies have shown that hexokinase (HXK) and Snf1-related kinases (SnRKs) participate in sugar sensing and signal transduction in plants. Diverse sugar signals can activate multiple HXK-dependent and HXK-independent pathways and use different molecular mechanisms to control transcription, translation, protein stability and enzymatic activity^37^. We detected differences in the abundance of hexokinase in mannan treated plants, which is likely due to mannans that were degraded into mannose monomers and were detected by hexokinase to regulate gene expression. Other parts of the mannan or mannose monomers may play a role in the membrane-dependent sugar signaling system, perhaps through the actions of G protein-coupled receptors or the mannan-binding lectin (MBL) pathway.

G protein-mediated signal transduction pathways play an important role in plant growth, development, regulation of secondary metabolism and other functions. It is generally recognized that a single extracellular receptor can activate many G proteins, amplifying the signal response, leading to a cascading effect. However, research in this area in plants is not as mature as in animals. In 1986, Dillenschneider demonstrated the existence of G proteins in the cell membrane of *Acer pseudoplatanus*, confirming for the first time the existence of G protein components in plants^38^. Other studies then showed the cellular localization of a G protein in duckweed and associated it with plant-specific light signal transduction^39^. In *Arabidopsis thaliana*, the G protein GPA1 can increase resistance to the fungal pathogen *Plectosphaerella cucumerina*, while AGB1 reduces resistance to fungal pathogens^40^. Similar results were also found in defense responses of GPA1 and AGB1 against the necrotic pathogens *Alternaria brassicicola* and *Fusarium oxysporum*^41^. These results illustrate that G proteins are involved in disease resistance in plants at the molecular level. G proteins, G protein-coupled receptors and G protein effectors constitute an important signaling system *in vivo*. Organisms and their cells need to quickly respond to very low concentration of outside stimuli, which generally occurs through G protein-coupled signal transduction.

When the mannan or mannose monomers in this study, as external signals, arrive at the cell membrane of *A. lancea*, they may act as ligands for the corresponding G protein receptor, which may include GDP-mannose-dependent α-(1–6)-phosphatidylinositol monomannoside mannosyltransferase, G-protein coupled receptor, mannosyltransferase, or the GTP binding signal recognition particle protein^42^,^43^,^44^. Following G protein-induced conformational change, forming tightly bound complexes, the following proteins may begin to be involved in the signal transduction: hypothetical protein COCSADRAFT_30752, RhoGEF, guanine nucleotide exchange factor for Rho/Rac/Cdc42-like GTPases^45^, protein synthesis factor GTP-binding protein, Rho GTPase-activating protein 19 isoform X5; following which, GDP and GTP are generated and used to give rise to the intracellular signaling molecule cAMP, after which several proteins may be phosphorylated: receptor-like protein kinase 3, adenylate kinase B, phosphoprotein, phosphoglycerate kinase, hexokinase.

Mounting evidence supports the importance of the MBL pathway of complement activation in the innate immune response of animals. MBL is an important receptor protein in this pathway that can selectively recognize the extracellular mannan or mannose residues, thereby activating the complement system^46^,^47^. MBL has also been reported to exist in plants^48^. Liénart *et al*. identified a 67 kDa membrane-bound chitosan specific lectin by chitosan-bead affinity chromatography from Rubus suspension cells. This protein may be a receptor for a chitosan resistance signaling molecule, whereby chitosan binds with its membrane receptor, triggering plant defense responses^49^. The crystal structures of two monocot mannan-binding lectins at 2 Å and 1.7 Å from *Narcissus pseudonarcissus* and *Scilla campanulata*, respectively, in the *Liliaceae* family were obtained by purification. The studies also showed that the monocot mannan-binding lectins can specifically combine with α-1,3 or α-1,6 glycosidic bond of mannan^50^,^51^, which shows that the unique structure of the mannan elicitor is necessary for interaction with the mannan lectin. In the present study, a critical receptor protein of the MBL pathway was identified. We can speculate, therefore, that specific immune processes depend upon mannan/mannose binding lectin molecules, which may occur after mannan enters *A. lancea* cells. It is worth mentioning that very few reports are available on the defense responses related to the MBL pathway in plants, and there is a lack clear evidence to support the role of MBL in this pathway. In this study, we discovered and validated a unique response mechanism of the MBL pathway to endophyte excreted mannan in *A. lancea*. These results are of great significance for understanding the immune response mechanisms of the plant MBL pathway. Volatile oils can be biosynthesized in large quantities to strengthen the immune function of the plant.

**Figure 7:**
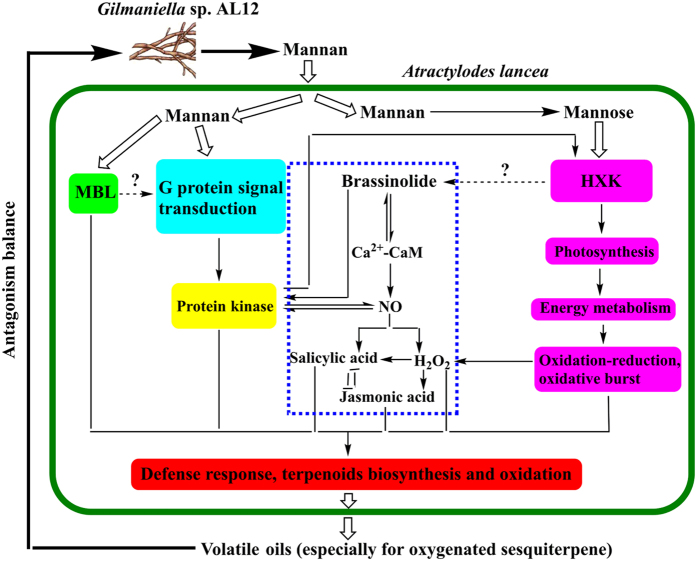
A mechanistic model of volatile oil accumulation in *A. lancea* following stimulation with mannan from the endophytic fungus *Gilmaniella* sp. AL12 (Hollow arrows represent material transport; filled arrows represent enzymatic reactions). Related proteins identified from *A. lancea* in this study: (1) MBL pathway (green color): Mannan-binding lectin. (2) G protein signal transduction pathway (sky-blue color): GDP-mannose-dependent alpha-(1–6)-phosphatidylinositol monomannoside mannosyltransferase, mannosyltransferase; Mannosyltransferase; GTP binding signal recognition particle protein; RhoGEF; Protein synthesis factor GTP-binding protein; Rho GTPase-activating protein 19 isoform X5; Adenylyl cyclase. (3) Protein kinase (yellow color): Receptor-like protein kinase 3; Adenylate kinase B; Phosphoprotein; Phosphoglycerate kinase. (4) Mannose pathway (carmine color): Hexokinase; Chlorophyll a/b binding protein; Ribulose bisphosphate carboxylase; ATP synthesis enzymes; Redox enzymes depend on NADH/NAD. (5) Terpenoids biosynthesis pathway (red color): Cytochrome P450; Epoxide hydrolase. The blue dotted box represents previous finding that AL12 could enhance volatile oil accumulation mediated by Ca^2+^-CaM, protein phosphorylation and many signalling molecules, such as NO, H_2_O_2_, salicylic acid, jasmonic acid, and brassinolide.

NO and H_2_O_2_ are important signalling molecules in stress responses^52^. After inoculation of *A. lancea* with the endophytic fungus AL12, our preliminary findings have shown that: (1) NO production depends on the salicylic acid and H_2_O_2_ signaling pathways, mediated by endophytic fungi induced volatile oil accumulation in the host plant. H_2_O_2_ can indirectly regulate the synthesis of salicylic acid in plants. The signaling molecule jasmonic acid is activated downstream of the NO and H_2_O_2_-mediated pathways, showing clear complementary with salicylic acid signaling in this process^2^,^11^. (2) Endophyte-induced Ca^2+^-CaM regulates the generation of NO and brassinolide, which was confirmed to be acting downstream of the brassinolide signal, leading to volatile oil accumulation^53^. (3) Phosphorylation is involved in endophytic-fungi induced volatile oil accumulation in *A. lancea*. The phosphorylation and NO pathways interact and the NO pathway may be located downstream of the brassinolide pathway^54^. Combining the above observations and the proteomics data obtained in this study, we present a mechanistic diagram of AL12-induced volatile oil accumulation in *A. lancea* (Fig. 7). Based on the model pattern, a conclusion can be draen that the endophyte AL12 secretes extracellular mannan to set up antagonistic balance in its interaction with *A. lancea*. One portion of the mannan can be degraded to mannose, which is detected by a membrane hexokinase that then regulates the expression of certain genes. Through the control of these genes, photosynthesis and energy metabolism are promoted. The activation of a series of oxidation-reduction reactions means more carbon metabolic flux would flow to terpenoid synthesis pathways. The other portion of the mannan can directly enhance autoimmunity and adaptability of the host *A. lancea* through G protein-mediated signal transduction and the MBL pathway. Volatile oil biosynthesis is activated in these reactions as a defense response. Several hormones, signaling molecules, and active oxygen burst have been confirmed to be involved in the above processes in our previous studies. It is also worth mentioning that endophyte-plant interaction or mannan regulation was more conducive to the accumulation of oxygenated sesquiterpenes, which is consistent with the data presented in Table 1.

In this paper, a novel exopolysaccharide mannan was discovered in an endophytic fungus of the medicinal plant *A. lancea*, and this mannan is apparently different from common polysaccharide elicitors that have been previously reported. More importantly, the novel mannan can significantly affect the synthesis of secondary metabolites in host. The mannan likely represents a key substance that the endophytic fungus AL12 specifically uses to induce the synthesis of volatile oils in the host *A. lancea*. This broadens our understanding of polysaccharides or carbohydrates as elicitors with a profound impact on specific plant metabolites. It also provides information on a systematic mechanism of interaction between host plants and their endophytes. Furthermore, as the mannan is secreted in abundant amounts in the extracellular milieu, it can be simply prepared from fungal fermentation. Consequently, this pure mannan elicitor could be used for large-scale production of active ingredients in *A. lancea*, greatly improving the yield of secondary metabolites, reducing the production cost, and bringing considerable economic and social benefits. It will also provide further information about the regulation of other valuable natural products in plants.

## Methods

### Plant materials and culture conditions

Meristem cultures of *A. lancea* were established as previously described^2^. Briefly, sterilized plantlets were maintained in Murashige and Skoog (MS) medium [supplemented with 0.3 mg.L^−1^ naphthaleneacetic acid (NAA), 2.0 mg.L^−1^ 6-benzyladenine, 30 mg.L^−1^ sucrose and 8% agar] in 100-mL Erlenmeyer flasks. The meristem cultures were then divided and transplanted into rooting medium (1/2 MS, 0.25 mg.L^−1^ NAA). The cultures were maintained in a growth chamber (25/18 °C day/night, with a light intensity of 80 μmol m^−2^ s^−1^ and a photoperiod of 12 h) and sub-cultured every 30 days. In order to compare the effect of *A. lancea* rhizome oil cavities (volatile oil storage organs in the rhizome) after AL12 exopolysaccharides treatment, microscopic observation of the oil cavities was analyzed as described previously^15^.

### Extraction of volatile oils and gas chromatography analysis

After each treatment, harvested plantlets were removed from agar and dried in a drying oven at 36 °C. After drying for at least 48 h, the plantlets were weighed every 2 h until a constant weight was reached. The method of Wang was employed to extract volatile oils from *A. lancea* plantlets^55^. The dried plants (1 g) were ground in liquid nitrogen and extracted in 4 mL cyclohexane for 10 h. After sonication and centrifugation, the supernatant was dried over anhydrous sodium sulfate and filtered through 0.22 μm membranes before analysis. Gas chromatography was carried out using a 7890 A gas chromatograph (Agilent, Santa Clara, CA, USA) equipped with a flame ionization detector. The temperature program and quantitative analyses of the main volatile components (β-caryophyllene, zingiberene, β-sesquiphellandrene, caryophyllene oxide, hinesol, β-eudesmol, atractylone, and atractylodin)^10^ were performed as previously described, with minor modifications. An Agilent DB-1HT (30 m × 0.32 mm × 0.10 μm) column was used with the following temperature program: the column was held at 100 °C for 4 min after injection, and was then increased by 10 °C.min^−1^ to 140 °C, held for 10 min, increased by 10 °C.min^−1^ to 220 °C, held for 10 min and increased by 10 °C.min^−1^ to 260 °C, held for 2 min. Nitrogen was used as a carrier at a flow rate of 0.8 mL.min^−1^. To measure the volatile oil levels, extraction and quantification were repeated three times per sample.

### Endophytic fungi and treatments

The endophytic fungus AL12 (*Gilmaniella* sp.) was isolated from the stems of *A. lancea*, cultured in PDA liquid medium, and incubated for 5 d at 28 °C on a shaker at 150 rpm. Each component was prepared as follows. Component A: The mycelium was homogenized with a glass pestle, diluted with double-distilled water (1:10, w/v), and treated by high pressure steam sterilization for 22 min at 121 °C. The crude elicitor preparation was obtained after sterilization; Component B: Component A was drained, resuspended in distilled water to wash, and this was step was repeated several times. The soluble cell substances were washed away, and then remaining sample was incubated for 12 h at 37 °C in combination with chitinase and β-1,3-glucanase. Component C: 3 L fermentation broth was filtered to remove mycelia and solid particles using a Buchner funnel. Lipophilic substances were removed by liquid-liquid extraction with ethyl acetate. The aqueous phase was destained with active carbon and the destaining solution was concentrated to 200 mL in a vacuum-rotary evaporator. Precooled 95% ethanol was added (5:1, v:v), stirred until white flocculent precipitates appeared, and the solution was stored at 4 °C overnight for stratification. The supernatant was discarded, and the precipitate was repeatedly washed with 95% ethanol, ethanol, and acetone. Finally, Component C (crude polysaccharide) was obtained after dehydration and removal of all proteins by the Sevag method. The same volume of aqueous solution or suspension (1 mg.mL^−1^) of Component A, B or C were sprayed onto *A. lancea* plantlets each week. The applied amount was 50 μL per plantlet. The basis for the quantity of elicitors’ sprayed onto the plants (50 μg) is the number of cells of *Gilmaniella* sp. AL12 found to colonize *A. lancea* in earlier experiments (data not shown). In the experiment using different concentrations of mannan, we set the amount of elicitor as 25, 50, 75 and 100 μg. Equal volumes of double distilled water were sprayed onto separate plants as control. Changes in volatile oil content in *A. lancea* plantlets were detected by the GC method after 20 days’ treatment.

### Purification of endophytic fungal polysaccharide

The crude polysaccharide (Component C) was dissolved in deionized water and purified by molecular sieve gel chromatography column (Sepharose CL-4B, 1.6 × 100 cm) using a chromatography system (ÄKTA^TM^ prime, GE Healthcare, USA). Operating conditions were as follows: the mobile phase was 0.15 moL.L^−1^ NaCl solution, with a flow rate of 0.2 mL.min^−1^. The polysaccharide fraction was collected and applied to ultrafiltration. The retentate was further purified by ion-exchange chromatography on a DEAE-Sepharose column (GE Healthcare, USA) equilibrated with 50 mM potassium phosphate buffer (pH 7.5). Elution was performed using a linear gradient of NaCl in the buffer. The collected eluent was applied with active carbon and dialysis (3.5 K MWCO). The purified polysaccharide was finally obtained as a white powder after freeze-drying. Total sugar concentration was determined by the phenol-sulfuric acid method^56^. The purity of the polysaccharide was measured by gel permeation chromatography (GPC) on a TSKgel G3000PWXL column (7.8 × 300 mm, Tosoh, Japan). The column was eluted with 0.3 M Na_2_SO_4_ (pH = 4.0) at a flow rate of 0.5 mL.min^−1^. The eluate was monitored by a refractive index detector (RID).

### Monosaccharide composition analysis of purified extracellular polysaccharide

The composition of polysaccharide monomers was determined by TLC and GC-MS. Preliminary polysaccharide composition analysis was performed using thin-layer chromatography (TLC) by a previously described method^57^. Sulfuric acid hydrolyzed monosaccharides from the purified extracellular polysaccharide were compared against analytical monosaccharide standards by TLC. Rhamnose, arabinose, mannose, glucose, fucose, and xylose were used as standards. Monosaccharide composition of the polysaccharide was further determined using a gas chromatography mass spectrometer (GC-MS, 7890A GC coupled with HP5975C MS, Agilent, Santa Clara, CA, USA). Each sample (10 mg) was hydrolyzed with 2 mol.L^−1^ trifluoroacetic acid (TFA) at 100 °C for 3 h. Excess acid was removed by 4 washes with methyl alcohol. The hydrolysates were reduced by NaBH_4_ (10 mg), and acetylated by pyridine (0.5 mL) and acetic anhydride (0.5 mL) at 40 °C for 2 h. The alditol acetates were dissolved in 2 mL methyl alcohol and filtered via a 0.45 μm filter membrane before injecting into the GC-MS with a DB-5 chromatographic column (0.2 mm × 35 m × 0.25 μm) for analysis. The temperature program was as follows: the initial temperature of column was 120 °C, and kept for 1 min, then increased to 210 °C at a rate of 30 °C.min^−1^, and kept for 5 min; N_2_ was used as the carrier gas and maintained at a flow rate of 15 mL.min^−1^. The temperature of mass spectrometer ion source was 250 °C.

### Structural characterization of the purified extracellular polysaccharide

MALDI-TOF MS (UltrafleXtreme, Bruker Daltonics, Germany) was used for the molecular weight calculation and distribution of the polysaccharide, with 2,5-dihydroxybenzoic acid (DHB) as the matrix^58^. FT-IR and NMR were used for the structural analysis of the polysaccharide. FT-IR: 5 mg sample of the polysaccharide was ground and pressed into a sheet with KBr and the FT-IR spectrum was obtained using a Thermo Nicolet NEXUS 670 FT-IR Mainframe at 500–4000 cm^−1^. NMR: 50  mg sample of the polysaccharide was dissolved in 0.5 mL D_2_O, and all spectra were measured at 25 °C. ^1^H NMR and ^13^C NMR experiments were performed using a nuclear magnetic resonance spectrometer (AVANCE 600, Bruker, Switzerland) at 600 MHz for 100 times and 151 MHz for 20000 times, respectively.

### Membrane protein extraction

Polysaccharide-treated and untreated control *A. lancea* plantlets were processed in parallel for membrane isolation according to a previously published protocol^16^ with some modification. One-week-old plantlets were collected, washed, weighed, and added to grinding buffer (Buffer A: 25 mM HEPES, 250 mM D-sorbitol, 10% glycerol, 0.6% PVPP, 5 mM ascorbic acid, 5 mM EDTA, 1 mM NaF, 1 mM sodium molybdate, 2 mM imidazole, 1 mM sodium orthovanadate, 5 mM DTT, 1 μM antiprotease, 1 μM Aprotinin, 1 μM leupeptin, 1 mM PMSF, pH7.5) at a ratio of 2 mL of grinding buffer/g of plant material. Plant leaves were homogenized using a high-speed homogenate instrument (IKA T10, Germany) at 20,000 rpm for 15 min on ice. The homogenate was filtered through 0.45 μm aquo system membranes and then centrifuged at 15,000 g for 10 min to remove cellular debris. Microsomal membranes were pelleted by ultracentrifugation (L8–60 M Ultracentrifuge, Beckman, Germany) at 100,000 g for 2 h. The pellet was resuspended in Buffer B (5 mM potassium dihydrogen phosphate, 250 mM D-sorbitol, 3 mM KCl, 0.1 mM DTT, 1 μM antiprotease, 1 μM Aprotinin, 1 μM leupeptin, 1 mM PMSF, pH 7.8) by pipetting up and down. Two-phase partitioning was performed using a solution containing 6.1% polyethylene glycol 3350, 6% Dextran T-500, and 8 mM KCl. After partitioning, the upper U3 phase was diluted with 10 volumes of Buffer B and centrifuged at 100,000 g for 2 h to collect membrane proteins. The pellet was resuspended in an appropriate amount of Buffer B without D-sorbitol by slowly pipetting up and down and then mixed with 5 volumes of TCA-acetone (13% TCA, 0.07% β-mercaptoethanol in 100% acetone) to precipitate the proteins. All the procedures were performed at 4 °C.

### Two-dimensional gel electrophoresis and image analysis

Precipitated proteins were pelleted by centrifugation at 15,000 g for 15 min. The supernatant was discarded and 1 mL of ice-cold acetone was slowly added to the pellet to remove any remaining salt. After 30 min on ice, the acetone was removed and the pellet was air-dried for 3 min before adding protein lysis buffer (7 M urea, 2 M thiourea, 2% CHAPS, 2% Triton X-100) to resolubilize the membrane proteins at room temperature 1 h^59^. Protein concentration was measured using the 2D-Quanti Kit (GE Healthcare, USA). For first dimension separation, 100 μg-samples were applied to Immobiline Dry Strips (24 cm, pH 3-10 NL, Bio-Rad, USA) using active rehydration at 30 V and 20 °C with an IPGphor III (GE Healthcare, Sweden) for 12 h. Subsequently, electrofocusing was carried out at 20 °C with a gradually increasing voltage as follows: 0–250 V, 1 h, Step; 250–1000 V, 2 h, Gradient; 1000–5000 V, 2.5 h, Gradient; 5000–10000 V, 4 h, Gradient; and to 80,000 Vhs with a maximum voltage of 10,000 V. Prior to SDS-PAGE to resolve the second dimension, the IPG strips were equilibrated with 15 mL equilibration buffer A (50 mM Tris-HCl, pH 8.8, 6 M urea, 30% (v/v) glycerol, 2% (w/v) SDS, 10 mg/mL DTT) at room temperature for 15 min, followed by equilibration in buffer B (as “A” but with 40 mg/mL iodoacetamide instead of DTT) for a further 15 min. The IPG strips were rinsed briefly with ddH_2_O and afterwards applied to 12.5–15% SDS-PAGE gradient gels (25.5 cm × 20.5 cm × 0.1 cm, Ettan^TM^ Daltsix system, GE Healthcare, Uppsala, Sweden). Second dimension separation according to molecular mass was performed at 1 W/gel and 20 °C for 1 h, followed by 15 W/gel and 20 °C for further 5 h until the bromophenol blue dye front had run to the bottom of the gel. Gels were subsequently stained using a modified silver staining protocol^60^. Gel images were captured, digitized (Image Scanner III, GE Healthcare, USA), and analyzed with Image Master 2D Platinum 7.0 (GE Healthcare, USA), including image filtering, background subtraction, normalization, and spot detection. Manual determination of spot positions was performed after automatic matching was complete. Spots with quantitative changes >2 and ANOVA values < 0.05 were considered as significantly altered between treatments.

### Protein identification using MALDI-TOF MS

Single protein spots were excised from the two-dimensional gel and subjected to in-gel digestion with trypsin (Promega, US). A 0.5 μL aliquot of the concentrated tryptic digest mixture in 0.1% TFA was mixed with 0.5 μL of CHCA matrix solution (5 mg/mL CHCA in 50% ACN/0.1% TFA) and spotted onto a freshly cleaned MALDI target plate. After air drying, the crystallized spots were analyzed using an ultrafleXtreme MALDI-TOF MS (Bruker Daltonics, Germany). Ionization was performed in MS and MS/MS by irradiation with a nitrogen laser (337 nm) operating at 1 kHz. Data were acquired at a maximum acceleration potential of 25 kV in positive and reflector modes. MS and MS/MS data were analyzed and peak lists were generated using flexAnalysis 3.1 (Bruker Daltonics, Germany). MS peaks between 850 and 3700 Da were selected and filtered to include those with a signal-to-noise ratio greater than 20. MS/MS peaks were selected based on a signal-to-noise ratio greater than 10. MS/MS spectra were search used to search against the NCBI non-redundant database with BioTools 3.2 (BioTools Europe Ltd., UK) using the MASCOT search engine (MatrixScience Ltd., UK), with a peptide and fragment ion mass tolerance of 100 ppm. High confidence identifications had statistically significant search scores (greater than 95% confidence, equivalent to MASCOT expect value p < 0.05) and accounted for the majority of ions present in the mass spectra.

### RNA extraction and quantitative RT-PCR

Total leaf RNA was extracted using TRIZOL reagent (Invitrogen, Carlsbad, CA)^41^. First-strand cDNA was synthesized from 1 μg of total RNA (PrimeScript One Step RT Reagent Kit; Takara, Dalian, China) and real-time qPCR was performed using the DNA Engine Opticon 2 Real-time PCR Detection System (Bio-Rad, Hercules, CA) and SYBR green probe (SYBR Premix Ex Taq system; Takara) with a 20.0 μL reaction volume that included: 10.0 μL SYBR Premix Ex Taq (2×), 0.4 μL PCR Forward Primer (10 mM), 0.4 μL PCR Reverse Primer (10 mM), 1.2 μL DNA template, 8.0 μL ddH_2_O. The thermal profile used consisted of an initial denaturation step at 95 °C for 90 s, followed by 40 cycles of 95 °C for 30 s, 57 °C for 30 s and 72 °C for 30 s. The qRT-PCR gene-specific primers were designed using Vector NTI 10.0 (Table 3). The gene encoding Actin (gi|9082316) was used as a reference for normalization. The qRT-PCR results were based on the average of triplicates and the standard deviation (SD) is shown.

### Statistical analysis

All experiments were performed at least three times, and the results are expressed as the mean ± standard deviation (n = 3). Data were analyzed using the Student’s t test. P values less than 0.05 were considered statistically significant.

## Additional Information

**How to cite this article**: Chen, F. *et al*. A novel exopolysaccharide elicitor from endophytic fungus *Gilmaniella* sp. AL12 on volatile oils accumulation in *Atractylodes lancea*. *Sci. Rep.* **6**, 34735; doi: 10.1038/srep34735 (2016).

## Supplementary Material

Supplementary Information

## Figures and Tables

**Table 1:** Concentration of volatile oils and their eight principal components after 20 days’ treatment by different components of AL12^a^.

Volatile oil (μg.g^−1^ DW)	Control	Component A	Component B	Component C	Purified EPS
β-Caryophyllene	31.25 ± 1.28 a	29.33 ± 1.35 a	32.02 ± 1.28 a	26.45 ± 1.21 a	24.15 ± 1.19 a
Zingiberene	21.75 ± 0.93 b	12.38 ± 0.37 a	20.67 ± 0.60 a	9.16 ± 0.38 a	6.24 ± 0.27 a
β-Sesquiphellandrene	15.69 ± 0.78 b	13.59 ± 0.39 a	16.12 ± 0.64 b	12.55 ± 0.49 a	10.03 ± 0.41 a
Caryophyllene oxide	18.06 ± 1.02 a	28.92 ± 1.21 a	19.21 ± 0.75 a	35.63 ± 1.50 b	42.71 ± 1.62 b
Hinesol	52.45 ± 2.28 a	143.76 ± 5.48 a	63.94 ± 2.24 b	148.14 ± 4.94 b	155.07 ± 5.06 a
β-Eudesmol	99.83 ± 4.96 a	221.47 ± 9.70 a	105.77 ± 4.34 a	230.60 ± 10.08 a	248.93 ± 10.13 a
Atractylone	5.57 ± 0.21 a	21.67 ± 1.07 a	7.22 ± 0.28 a	32.41 ± 1.36 a	35.41 ± 1.42 a
Atractylodin	138.96 ± 5.87 a	139.47 ± 5.30 b	141.56 ± 5.38 a	152.58 ± 5.80 b	162.73 ± 6.17 b
Total essential oils	383.56 ± 15.26 a	610.59 ± 21.37 a	406.51 ± 16.67 a	647.52 ± 27.20 a	685.27 ± 24.38 a

^a^Values are means from three biological replicates with the corresponding standard deviations. Values followed by different lowercase letters are significantly different according to Tukey’s multiple-comparison test. Control was the experimental group that was not treated with any component. Component A was the sterilized crude mycelium of AL12, Component B was the chitinase and β-1,3-glucanase hydrolyzed cell wall products of AL12, and Component C was the crude extracellular polysaccharides derived from the AL12 fermentation broth. Purified EPS was purified Component C.

**Table 2:** Identification of differentially expressed proteins between control *A. lancea* plantlets and plantlets induced by AL12 exopolysaccharides.

Putative function	Spot no.^a^	Protein	Gene or gene locus	Mascot score	Protein ID	Mr (Da)^b^	pI^b^	Fold change^c^	p-value
Photosynthesis	4	hypothetical protein PHAVU_007G155500g	PHAVU_007G155500g	167	gi|593687543	14047	9.60	2.87	0.031
	6	chlorophyll a/b-binding protein CP26 precursor	LOC_Os11g13890	152	gi|62733870	24317	5.95	4.35	0.029
	14	chlorophyll a/b binding protein precursor	AF220527	162	gi|6716783	28542	5.29	2.58	0.047
	17	chlorophyll a/b binding protein	CAB1	238	gi|5714656	28613	5.29	3.21	0.023
	21	chlorophyll a-b binding protein 8	LOC100232927	158	gi|225436257	29511	7.85	2.06	0.036
	35	carbonic anhydrase	FBU08398	136	gi|40737972	36025	5.85	7.98	0.041
	37	ribulose bisphosphate carboxylase large chain	rbcL	305	gi|131899	52159	6.13	10.12	0.040
	58	ribulose-1,5-bisphosphate carboxylase/oxygenase large subunit, partial	rbcL	199	gi|1881503	52231	6.03	3.51	0.019
	59	ribulose-1,5-bisphosphate carboxylase, partial	rbcL	188	gi|2961313	53408	6.05	2.01	0.035
Oxidation-reduction, oxidative burst	2	hypothetical protein GUITHDRAFT_152572, NADH-ubiquinone reductase complex 1 MLRQ subunit	GUITHDRAFT_152572	84	gi|551660038	12249	9.95	10.29	0.021
	20	putative protein, NADPH-dependent FMN reductase	AT4g27270	64	gi|3269288	22355	6.30	4.32	0.048
	24	oxygen-evolving enhancer protein 2	LOC103990328	146	gi|695040260	28178	8.61	2.02	0.022
	25	photosystem II oxygen-evolving enhancer protein 2	GSCOC_T00013178001	258	gi|661878401	28714	8.22	2.14	0.031
	30	N-ethylmaleimide reductase	FadH	74	gi|659891198	39341	4.94	2.16	0.037
	34	monooxygenase	AT4G38540	69	gi|3426064	45505	5.94	2.53	0.020
	42	NAD-dependent epimerase/dehydratase	MtrDRAFT_AC155890g8v2	81	gi|124360315	31765	7.64	2.23	0.034
	45	NADPH: adrenodoxin	NGA_0622900	84	gi|553192841	153673	8.64	0.47	0.028
	46	gamma-glutamyl putrescine oxidoreductase, putative, partial	RCOM_0368500	82	gi|223524108	29290	7.85	2.28	0.029
	47	oxygen-evolving enhancer protein 1	PSBO	190	gi|131385	35595	5.84	3.74	0.019
	48	hypothetical protein BCCGELA001_06795, partial, COG1032 Fe-S oxidoreductase	BCCGELA001_06795	70	gi|404270905	35764	6.13	3.62	0.027
	55	NADP-dependent oxidoreductase P1	LOC100282970	88	gi|226528403	38936	6.22	2.29	0.024
Energy metabolism	8	adenylate kinase B	ADK-B	72	gi|728811055	27013	6.45	2.44	0.012
	13	ATP synthase delta chain	LOC104229705	230	gi|698456786	26776	8.94	3.93	0.044
	18	butyrate kinase	PRK03011	84	gi|499737230	39210	5.81	0.42	0.045
	19	protein phosphatase 2A catalytic subunit	PP2Ac2	74	gi|350537893	35010	4.78	2.11	0.039
	32	phosphoglycerate kinase	LOC100249576	111	gi|225464995	50166	8.26	8.20	0.025
	33	ATP synthase gamma chain	LOC103446721	135	gi|657983988	41501	7.52	3.33	0.031
	52	V-type proton ATPase catalytic subunit A	AT1G78900	94	gi|332198050	69077	5.29	10.58	0.038
	53	ATP-dependent Clp protease	MTR_3g098310	72	gi|657395325	88029	7.91	2.90	0.030
	56	ATP synthase beta subunit, partial	atpB	630	gi|6706178	52616	4.99	11.02	0.016
	57	AtpB	atpB	143	gi|682124547	53618	5.13	2.12	0.025
	60	hexokinase	HXK1	115	gi|110740344	53707	5.76	+∞	0.034
	62	ATP synthase CF1 alpha subunit	atpA	253	gi|313183946	55759	5.62	2.10	0.042
Environmental stress response	3	C2.6 protein, Universal stress protein family	C2.6	64	gi|33307140	18263	5.50	4.24	0.029
	11	chaperone protein dnaJ	MTR_2g033460	68	gi|357448821	81171	9.20	6.22	0.016
	41	heat shock protein SSB1	MGG_11513	82	gi|389641395	66853	5.38	3.07	0.027
	49	protein enhanced disease resistance 2	LOC101503113	60	gi|502151333	83877	6.52	7.28	0.031
	54	putative disease resistance protein RGA1	LOC104447499	67	gi|702359424	43719	5.94	6.78	0.033
	61	unnamed protein product, Resistant to P. syringae 6	TRAES_3BF089300150CFD_c1	61	gi|669031836	91753	8.00	3.44	0.041
Signal transduction	10	hypothetical protein COCSADRAFT_30752, RhoGEF, guanine nucleotide exchange factor for Rho/Rac/Cdc42-like GTPases	COCSADRAFT_30752	63	gi|628085938	32647	10.25	2.42	0.039
	16	mannan-binding lectin	AF347116	81	gi|13517976	28118	5.74	+∞	0.030
	36	GDP-mannose-dependent alpha-(1–6)-phosphatidylinositol monomannoside mannosyltransferase	pimB	93	gi|664282737	40669	9.07	+∞	0.024
	38	G-protein coupled receptor 1, partial	KM399174	84	gi|700256424	33729	9.38	3.31	0.041
	66	protein synthesis factor GTP-binding protein	HACJB3_RS04355	91	gi|495689754	58532	5.00	5.34	0.017
	72	adenylyl cyclase	AT3G21465	60	gi|11994381	44065	9.07	8.61	0.019
	75	TonB-linked outer membrane protein, SusC/RagA family	BACCELL_02879	66	gi|224520396	126159	5.84	4.62	0.028
	76	spectrin alpha chain-like isoform 2	LOC100874878	80	gi|383848576	280882	5.14	2.97	0.044
	77	GPI mannosyltransferase 2	F775_16289	78	gi|475571170	56255	9.30	5.14	0.034
	78	rho GTPase-activating protein 19 isoform X5	ARHGAP19	80	gi|114632111	52589	9.35	+∞	0.025
	81	receptor-like protein kinase 3	M569_06486	106	gi|527201051	68638	8.95	7.69	0.029
	82	GTP binding signal recognition particle protein	ffh	60	gi|732152276	56257	9.11	+∞	0.031
Cytoskeletal	43	actin	AF282624	203	gi|9082317	41934	5.64	2.80	0.038
	44	actin-2	LOC105059495	271	gi|9082316	41646	5.23	3.58	0.025
Amino acid metabolism	5	alpha chain of nascent polypeptide associated complex	NbNACa1	80	gi|124484511	21911	4.32	0.26	0.026
	7	serine racemase	LOC103705524	66	gi|672128011	35616	6.01	3.00	0.039
	12	glutamate 5-kinase	MTR_5g042980	81	gi|357487315	34715	5.86	0.47	0.031
	15	inner membrane ABC transporter, putative	RCOM_1816900	64	gi|255605404	33486	9.04	0.19	0.018
	22	glutamine synthetase precursor	plGS	101	gi|5733730	48097	8.34	3.63	0.015
	28	ABC transporter C family member 3	MRP3	69	gi|42572407	124952	6.40	5.45	0.029
	29	phenylalanine ammonia lyase	PAL	71	gi|58618144	77459	5.83	0.37	0.032
	39	Glutamate-ammonia-ligase adenylyltransferase, Bifunctional glutamine-synthetase adenylyltransferase/deadenyltransferase	glnE	63	gi|430009452	111444	5.97	0.16	0.033
	50	predicted protein, glutaminyl transferase	PHYPADRAFT_184130	73	gi|168024584	126285	6.81	2.46	0.037
Sugar transportation	19	sugar isomerase domain-containing protein	ARALYDRAFT_485874	77	gi|297322103	37783	6.51	5.45	0.031
	23	sugar ABC transporter ATP-binding protein	yphE	74	gi|501396496	28244	7.03	2.47	0.024
	31	monosaccharide ABC transporter ATP-binding protein, CUT2 family	Chro_1853	85	gi|428008801	55504	5.35	3.45	0.041
Plant secondary metabolism	26	putative oxysterol-binding protein	AWRI1499_2309	63	gi|385303702	43378	8.78	4.03	0.046
	40	cytochrome P450	AT4g19230	76	gi|7268718	52437	8.74	2.10	0.048
	64	storage protein	AY439332	165	gi|37789212	61803	5.52	0.20	0.019
	67	epoxide hydrolase	MTR_7g034950	65	gi|357504133	42877	5.95	3.41	0.029
Transcription and translation	1	60S acidic ribosomal protein P3	POPTR_0009s03780g	62	gi|224106463	11941	4.22	6.56	0.031
	9	ribonuclease	MTR_4g095410	63	gi|357476363	41332	8.72	5.37	0.029
	51	helicase, transcription/DNA replication, recombination, and repair	CF70_027305	90	gi|645573106	76855	5.78	0.41	0.027
	63	transcription factor 20	TCF20	69	gi|395540747	203758	8.84	0.38	0.019
	68	inhibitor of nuclear factor kappa-B kinase subunit beta	ikbkb	79	gi|734610408	86822	6.15	2.19	0.012
	69	RNA polymerase sigma factor sigD	LOC18440908	67	gi|586748416	50552	10.01	3.32	0.023
	70	hypothetical protein VITISV_028576, chromosome segregation protein SMC	VITISV_028576	68	gi|147818418	112276	5.01	2.25	0.031
	73	acetyltransferase, N-Acyltransferase superfamily	EL17_00675	82	gi|660633913	25375	5.61	3.24	0.048
Metabolism of cofactors	65	formate-tetrahydrofolate ligase	PHEL85_2785	63	gi|697005335	60833	8.54	0.14	0.041
	71	CoA-transferase	CaiB	85	gi|666644607	42162	5.64	2.96	0.025
	80	4-phosphopantetheinyl transferase	DA73_94860	60	gi|692202221	23823	6.09	2.38	0.027
Others	27	outer membrane assembly lipoprotein YfgL	YfgL	61	gi|846382982	41113	9.13	3.15	0.036
	74	lysophospholipase	Cha6605_1617	61	gi|428016673	32143	6.84	4.10	0.028
	79	hypothetical protein, predicted periplasmic protein	—	69	gi|575528046	53794	7.64	2.98	0.014
	83	membrane protein	—	77	gi|739293224	27839	6.12	10.24	0.036

^a^Spot numbers refer to the proteins labeled in Fig. 4.

^b^Discrepancies exist between the measured and the predicted proteins due to modification or degradation.

^c^The values are fold change of proteins in *A. lancea* induced by AL12 exopolysaccharides compared to control *A. lancea* plantlets.

**Table 3:** Primers used in this study.

Gene^a^	Sequence Length	Product Length	Sense Primer (5′-3′)	Anti-sense Primer (5′-3′)
*actin*	1134	441	TATGGTTGGTATGGGACA	ATCAGTGAGTCGGTAAGGT
*cab1*	986	420	GTCCTAATGGGATTCGTCG	TTCGCAAAGGTCTGTCTGTT
*rbcL*	1411	391	CTTCACATTCACCGTGCRAT	GATTCGCTACANNACCTG
AT4G38540	1410	420	AGTACCGACCTCCATGGGAA	ATCATGGGACAAGGCTTCCG
LOC100282970	1302	411	TCAAGACCAGGTTCGGCTTC	GGAAGAGTCCTATGAGCGCC
*atpB*	1497	331	TATTGCCAAAGCTCACGG	ATAACCCACAGCGGAAGG
*atpA*	1527	401	GGCAGGTGAGCTGGTTACAT	TATCGGTTGCCACTGCTGTT
MGG_11513	2328	382	CAACTCTGTGGGCAAGCT	AAAGACGGGCAGGTAAGT
LOC104447499	1152	462	CCGTCGCAATTTACAGCATCAA	CCTCAGGTACACACACCCAT
AT3G21465	1425	310	AGGACGCCAAACTGATGGAT	AGCAGAAAACCCTTTGCACAT
ARHGAP19	523	439	AACCGCCGCAAGATGACA	GCGACAGGGACTGGTAGACG
*ffh*	5382	404	AAATTCGTTTGCGTAGGT	AAGGCATGTGCATGTGAT
*HXK1*	2030	439	AATGGCATGGTCTGCTTCCA	ATTCCAGCAGCAGAGAGACG
AF347116	864	409	CCGACAACCAGCTCTCCTTC	ACCAGGCTGTAGTCTCTCGT
AT4g19230	1435	479	GCGAGTGTGATGTCGTGGAT	CTCGCTCCAACAATTGACCA
MTR_7g034950	1173	353	GTCATTGCTGCTGCTTCAGG	CCTGGTGGCCCTGTTACAAT

^a^The gene encoding Actin (*actin*) was used as a reference for normalization. Gene names of related proteins: Chlorophyll a/b binding protein (*cab1*); Rbulose-1,5-bisphosphate carboxylase/oxygenase (*rbcL*); NADP-dependent oxidoreductase (LOC100282970); Monooxygenase (AT4G38540); ATP synthase beta subunit (*atpB*); ATP synthase CF1 alpha subunit (*atpA*); Heat shock protein SSB1 (MGG_11513); Putative disease resistance protein RGA1 (LOC104447499); Adenylyl cyclase (AT3G21465); Rho GTPase-activating protein 19 isoform X5 (ARHGAP19); GTP binding signal recognition particle protein (*ffh*); Hexokinase (*HXK1*); Mannan-binding lectin (AF347116); Cytochrome P450 (AT4g19230); Epoxide hydrolase (MTR_7g034950).
