# Supplementary material for: A novel exopolysaccharide elicitor from endophytic fungus Gilmaniella sp. AL12 on volatile oils accumulation in Atractylodes lancea
Source: Sci Rep. 2016 Oct 5;6:34735. doi: 10.1038/srep34735 (PMC5050437; doi:10.1038/srep34735)
Supplement: Supplementary Information [file srep34735-s1.pdf]

Supplementary Information

A novel exopolysaccharide elicitor from endophytic fungus *Gilmaniella* sp. AL12 on volatile oils accumulation in *Atractylodes lancea*

Fei Chen[#], Cheng-Gang Ren[#], Tong Zhou, Yu-Jia Wei & Chuan-Chao Dai[*]

Jiangsu Key Laboratory for Microbes and Functional Genomics, Jiangsu Engineering and Technology Research Center for Industrialization of Microbial Resources, College of Life Sciences, Nanjing Normal University, Nanjing 210023, China.

[#]Fei Chen and Cheng-Gang Ren contributed equally to this work.

[*]Corresponding authors:

Chuan-Chao Dai, Tel/Fax: +86-25-85891382, E-mail: daichuanchao@njnu.edu.cn.

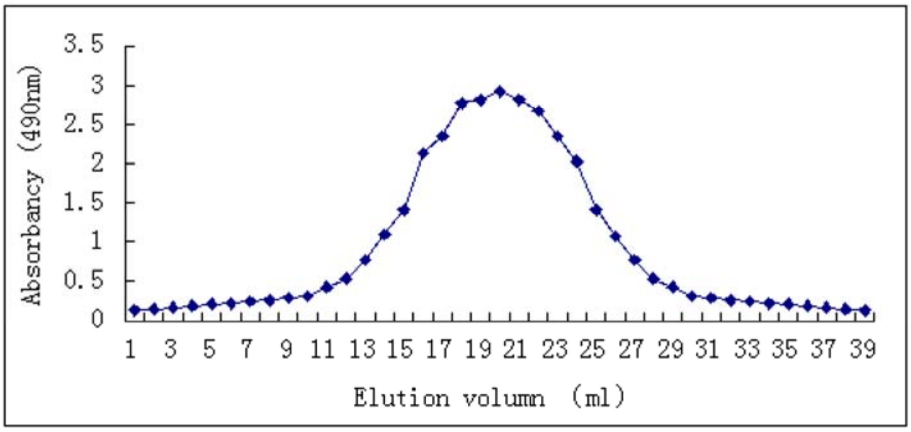

Figure S1. The elution curve of crude polysaccharides isolated from *Gilmaniella* sp. AL12 on a molecular sieve gel chromatography column (Sepharose CL-4B, 1.6 × 100 cm) eluted with the 0.15 moL.L^{-1} NaCl aqueous solutions at a flow rate of 0.2 mL.min^{-1}.

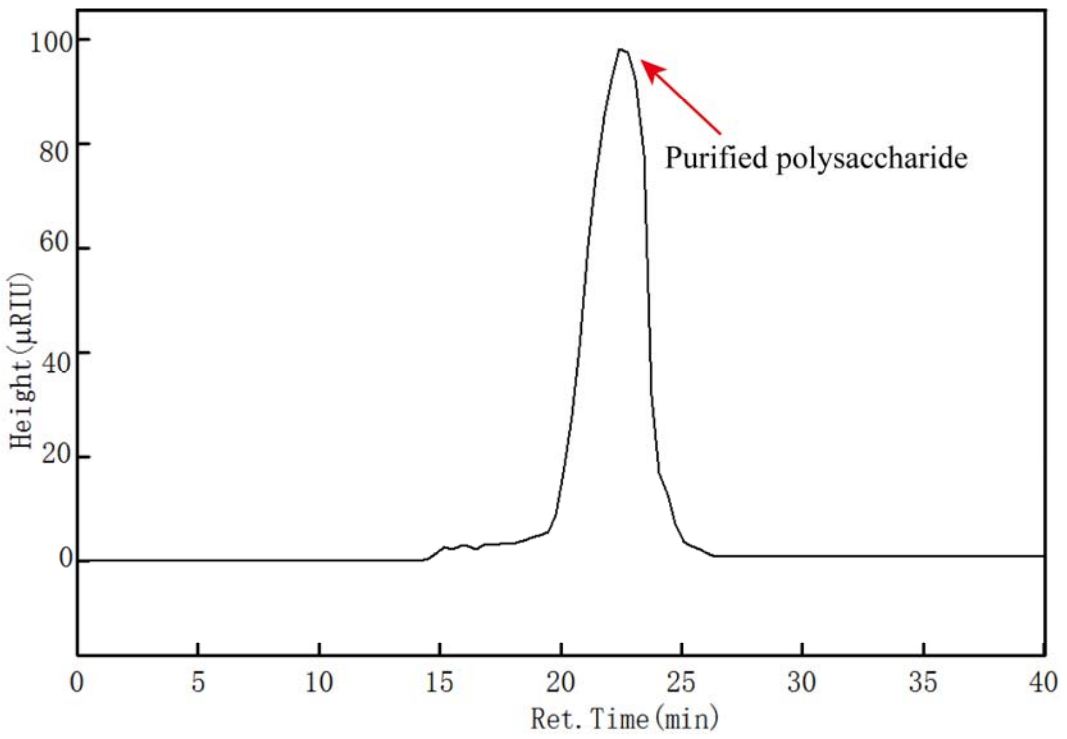

Figure S2. The purity identification of the polysaccharide from *Gilmaniella* sp. AL12 was measured by gel permeation chromatography (GPC) on a TSKgel G3000PWXL column.

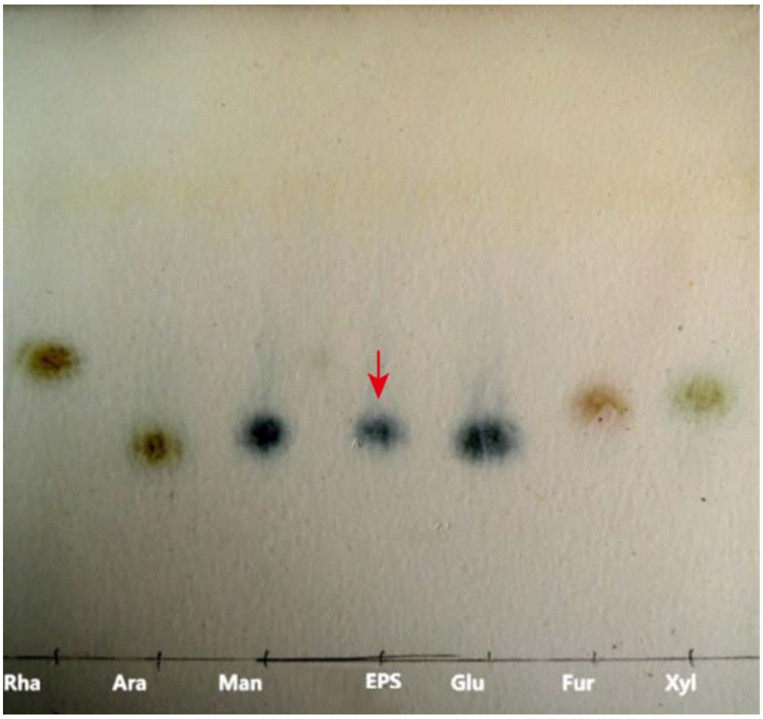

Figure S3. TLC results of hydrolyzed monosaccharide and standard monosaccharides. The Rf value and color of monosaccharide: EPS-0.29, dark blue. Rha-0.35, brown. Ara-0.276, brown. Man-0.29, dark blue. Glu-0.278, dark blue. Fur-0.32, brown. Xyl-0.33, green. EPS stands for hydrolyzed monosaccharide of purified exopolysaccharide from *Gilmaniella* sp. AL12, Rha stands for rhamnose standard monosaccharide, Ara stands for arabinose standard monosaccharide, Man stands for mannose standard monosaccharide, Glu stands for glucose standard monosaccharide, Fur stands for fucose standard monosaccharide. Xyl stands for xylose standard monosaccharide.

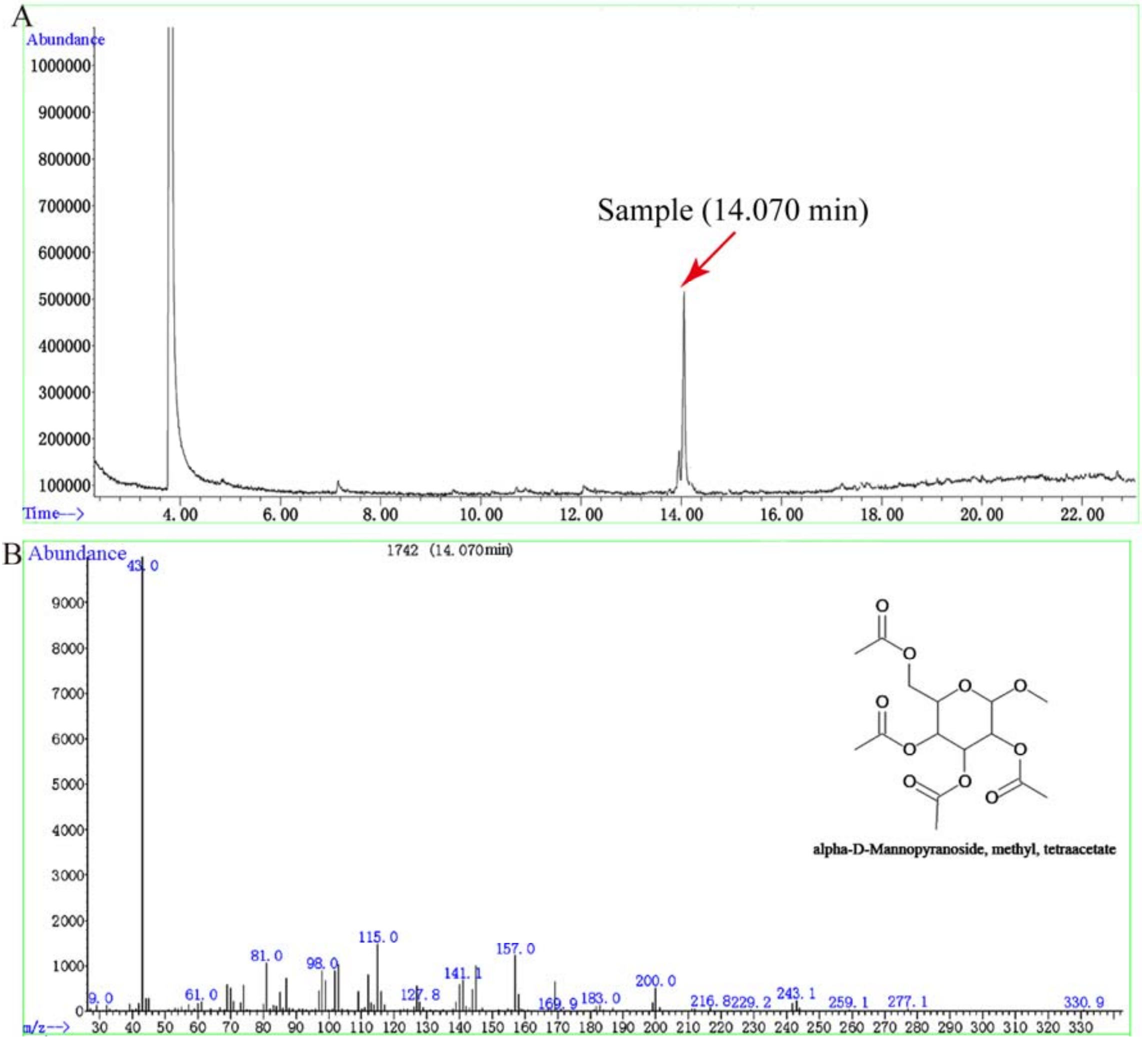

Figure S4. The GC-MS result of monosaccharide derivant from *Gilmaniella* sp. AL12 purified exopolysaccharide. (A) Total ion gas chromatogram of sample; (B) Mass spectrogram of sample.

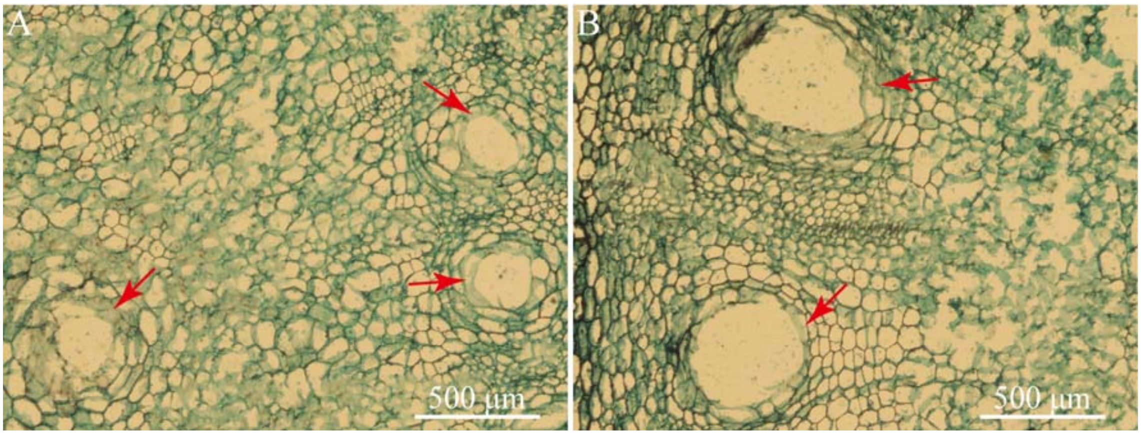

Figure S5. The effect of *A. lancea* rhizome oil cavities by *Gilmaniella* sp. AL12 exopolysaccharides treatment. (A) The control group of which any component was not injected; (B) The experiment group of purified extracellular polysaccharides treated. The red arrows represent *A. lancea* rhizome oil cavities, they are volatile oils storage organs in rhizome of *A. lancea* plantlets.
